# Association between early spontaneous abortion and homocysteine metabolism

**DOI:** 10.3389/fmed.2024.1310112

**Published:** 2024-03-25

**Authors:** Fangliang Lei, Lili Zhang, Li Wang, Wentao Wu, Fei Wang

**Affiliations:** ^1^Office of Hospital Infection Management, Shaanxi Provincial People’s Hospital, Xi’an, China; ^2^Department of Epidemiology and Health Statistics, School of Public Health, Xi’an Jiaotong University Health Science Center, Xi’an, China; ^3^Center of Health Examination, Shaanxi Provincial People’s Hospital, Xi’an, China; ^4^Department of Gynecology, Shaanxi Provincial People’s Hospital, Xi’an, China; ^5^School of Life Sciences, Northwestern Polytechnical University, Xi’an, China

**Keywords:** spontaneous abortion, homocysteine (HCY), folate, vitamin B_12_, MTHFR 677 gene

## Abstract

**Objective:**

The purpose of this study is to explore the effects of homocysteine (HCY) metabolism and related factors on early spontaneous abortion.

**Methods:**

We conducted a hospital-based case–control study and included a total of 500 cases and 1,000 controls in Shaanxi China. Pregnant women waiting for delivery in the hospital were interviewed to report their characteristics and other relevant information during pregnancy. The unconditional Logisitic regression model was applied to assess the association between early spontaneous abortion and HCY metabolism and related factors. The multiplicative model was applied to assess the effects of interaction of HCY metabolism and related factors on early spontaneous abortion. The logit test method of generalized structural equation model (GSEM) was used to construct the pathway diagram of HCY metabolism and related factors affecting early spontaneous abortion.

**Results:**

Folic acid supplementation and adequate folic acid supplementation during periconception were the protective factors of early spontaneous abortion (OR = 0.50, 95% CI: 0.38–0.65; OR = 0.44, 95% CI: 0.35–0.54). The serum folate deficiency, higher plasma HCY in early pregnancy, the women who carried the MTHFR 677TT genotype were the risk factors of early spontaneous abortion (OR = 5.87, 95% CI: 1.53–22.50; OR = 2.94, 95% CI: 1.14–7.57; OR = 2.32, 95% CI: 1.20–4.50). The women’s educational level and maternal and child health care utilization affected the occurrence of early spontaneous abortion by influencing the folic acid supplementation during periconception. The folic acid supplementation during periconception affected the occurrence of early spontaneous abortion by influencing the level of serum folate or plasma HCY in early pregnancy. The maternal MTHFR 677 gene polymorphism affected the occurrence of early spontaneous abortion by influencing the level of serum folate in early pregnancy. In terms of the risks for early spontaneous abortion, there was multiplicative interaction between higher plasma HCY in early pregnancy, serum folate deficiency in early pregnancy and maternal MTHFR 677TT genotype (OR = 1.76, 95% CI: 1.17–4.03), and there was multiplicative interaction between higher plasma HCY and serum folate deficiency in early pregnancy (OR = 3.46, 95% CI: 2.49–4.81), and there was multiplicative interaction between serum folate deficiency in early pregnancy and maternal MTHFR 677TT genotype (OR = 3.50, 95% CI: 2.78–5.18). The above interactions are all synergistic. The occurrence risk of early spontaneous abortion was significantly increased if multiple factors existed at the same time.

**Conclusion:**

Our study is the first time to construct the pathway of HCY metabolism and related factors affecting early spontaneous abortion, and provides a comprehensively new idea to prevent and reduce the occurrence of spontaneous abortion.

## Introduction

Spontaneous abortion refers to the process of automatically separating an embryo from the mother due to certain pathological factors without using any artificial methods. It generally occurs before the 28th week of pregnancy. The definition of spontaneous abortion varies among countries and international organizations, affecting estimations of the risk and prevalence of spontaneous abortion. In 1957, the World Health Organization (WHO) defined spontaneous abortion as a pregnancy that was less than 28w pregnant and had a weight of less than 1,000 g that was terminated due to non human factors. In 1977, the WHO revised its definition to include a pregnancy in which a non viable fetus is delivered at less than 20w gestation and weighs less than 500 g (1). The American Society for Reproductive Medicine defines miscarriage as a clinical pregnancy loss of less than 20 weeks of gestation (2). The European Society of Human Reproduction and Embryology defines miscarriage as the loss of pregnancy before 22 weeks of gestation (3). According to the time of occurrence, spontaneous abortion can be divided into early abortion (<12w) and late abortion (≥12w and < 28w), with early spontaneous abortion accounting for more than 80% of all spontaneous abortions (4).

The incidence of spontaneous abortion is approximately 10 to 25% among clinically confirmed pregnancies (5, 6), and appears to be increasing year by year. Spontaneous abortion has adverse effects on both physiology and psychology of women of childbearing age. The consequences of miscarriage are both physical, such as bleeding or infection, and psychological. Psychological consequences include increases in the risk of anxiety, depression, post-traumatic stress disorder, and suicide. Miscarriage, and especially recurrent miscarriage, is also a sentinel risk marker for obstetric complications, including preterm birth, fetal growth restriction, placental abruption, and stillbirth in future pregnancies (7, 8), and a predictor of longer-term health problems, such as cardiovascular disease and venous thromboembolism (9).

**Figure 1:**
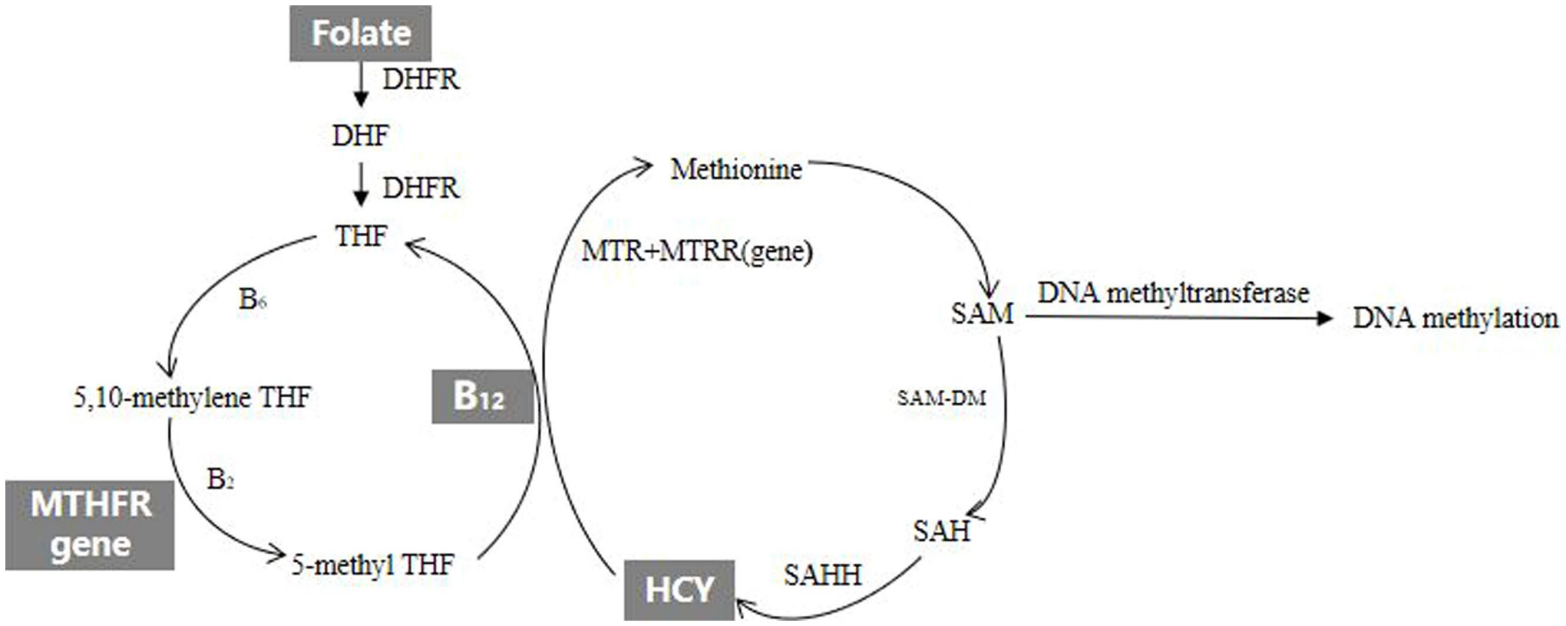
The diagram of HCY metabolism.

However, the etiology of spontaneous abortion remains unknown. Previous studies have reported that both genetic (10, 11) and environmental (12–14) factors may increase spontaneous abortion risk. Existing evidence suggests that some factors related to HCY metabolism, including folate, vitamin B_12_ and MTHFR 677 genotype are associated with spontaneous abortion. The diagram of HCY metabolism is shown in Figure 1, and the explanation of relevant English abbreviations can be found in Supplementary Table S1. The prospective cohort study named Nurse’s Health Study-II in America found that higher intake of folate from supplements was associated with reduced risk of spontaneous abortion (15). A study in North India reported that vitamin B_12_ deficiency was associated with an increased risk of recurrent spontaneous abortion (16). Another study from China found that MTHFR could affect sperm DNA integrity through affecting DNA methylation, which led to an increase in the rate of early spontaneous abortion in spouses (17). In practice, HCY metabolism and related factors exist high degree of intercorrelation. Studies on individual factor of HCY metabolism can hardly consider these complex interactions. The pathway analysis offers a comprehensive method to examine the relationships between HCY metabolism and spontaneous abortion. To our knowledge, there have been no published studies comprehensively evaluating the effects of HCY metabolism and related factors on spontaneous abortion. This case–control study aimed to explore the effects and pathways of HCY metabolism and related factors on early spontaneous abortion.

## Methods

### Study design and participants

We conducted a case–control study in three tertiary comprehensive hospitals in Xi’an City, Shaanxi Province, China from November 2016 to December 2018. Cases and controls were recruited among the pregnant women who sought medical advice or were waiting for delivery in the obstetrics and gynecology departments and who resided in Shaanxi Province during pregnancy. The women with gestational age less than 12 weeks and diagnosed with spontaneous abortion by b-ultrasound or electrochemical luminescence detection of serum human chorionic gonadotropin (HCG) were included in the case group. The women who gave birth to normal full-term infants and had not been found abnormal fetal development during the prenatal examination were included in the control group. Mothers with diabetes or multiple gestations were excluded from the study because of potentially different etiologies. The ratio of the number of controls and cases included in the same time frame in the same three hospitals was 2:1.

Further detailed information about the sample size calculations are provided in e-component Supplementary Method section “sample size calculations”. The final analysis included 500 cases and 1,000 controls who had completed the questionnaires.

The study was conducted in accordance with the Declaration of Helsinki, and the Xi’an Jiaotong University Health Science Center approved it (approval code: 2012008). All women gave written informed consents.

### Biomarkers measurement

The related blood biochemical indicators including the concentrations of folate, vitamin B_12_, HCY and MTHFR 677 genotype were measured routinely when pregnant women were in hospital, and these values were extracted from the medical records.

### Covariables assessment

A face-to-face questionnaire survey was used to collect relevant information of the women. The study information included pregnancy outcome, socio-demographic characteristics, environmental risk factors, reproductive history, sickness status during periconception, maternal and child health care utilization and nutrients supplementation during periconception. Folic acid supplementation during periconception was defined as taking folic acid (0.4 g/d) for 30 days or more during periconception. Adequate folic acid supplementation during periconception was defined as taking folic acid (0.4 g/d) for 90 days or more during periconception. Serum folate deficiency was defined as concentration of serum folate of peripheral blood in women during the first trimester for <3 ng/mL (18). Serum vitamin B_12_ deficiency was defined as concentration of serum vitamin B_12_ of peripheral blood in women during the first trimester for <200 pg/mL (19). Higher plasma HCY was defined as concentration of plasma HCY of peripheral blood in women during the first trimester for >15 μmol/L (20).

### Statistical analysis

The unconditional Logisitic stepwise forward regression model was applied to assess the association between early spontaneous abortion and HCY metabolism and related factors. The multiplicative model was applied to assess the effects of interaction of HCY metabolism and related factors on early spontaneous abortion. The logit test method of generalized structural equation model (GSEM) was used to construct the pathway diagram of HCY metabolism and related factors affecting early spontaneous abortion. All statistical analyses were performed using the Stata software (version 12.0; StataCorp, College Station, TX, USA). The tests were two-tailed with *p* < 0.05 being considered statistically significant.

## Results

### Sample characteristics

The general characteristics of the study sample are shown in Table 1. Maternal education, residence, household wealth index, gravidity, environmental risk factors, sickness status during periconception and maternal and child health care utilization were significantly different between the two groups. No significant differences were found in maternal age or parity between the two groups. Mothers who were included in the subgroup for the assessment of HCY metabolism-related biomarkers and mothers who were not included in the subgroup showed comparable general characteristics (Supplementary Table S2).

**Table 1:** Characteristics of the study sample.

Characteristics	Case [*n* (%)] (*N* = 500)	Control [*n* (%)] (*N* = 1,000)	*p*
Maternal age			0.248
<30 years	310 (62.00)	650 (65.00)	
≥30 years	190 (38.00)	350 (35.00)	
Maternal education			<0.001
Senior high school or below	215 (43.00)	250 (25.00)	
College degree or above	285 (57.00)	750 (75.00)	
Residence			<0.001
Rural	326 (65.20)	311 (31.10)	
Urban	174 (34.80)	689 (68.90)	
Household wealth index			<0.001
Poor	263 (52.60)	280 (28.00)	
Moderate or above	237 (47.40)	720 (72.00)	
Environmental risk factors during pregnancy			<0.001
Yes	328 (65.60)	479 (47.90)	
No	172 (34.40)	521 (52.10)	
Gravidity			0.004
1	147 (29.40)	370 (37.00)	
≥2	353 (70.60)	630 (63.00)	
Parity			0.854
0	259 (51.80)	513 (51.30)	
≥1	241 (48.20)	487 (48.70)	
Sickness status during periconception			<0.001
Yes	364 (72.80)	582 (58.20)	
No	136 (27.20)	418 (41.80)	
Maternal and child health care utilization			<0.001
Worse	446 (89.20)	772 (77.20)	
Better	54 (10.80)	228 (22.80)	

### The association between early spontaneous abortion and HCY metabolism and related factors

The association between early spontaneous abortion and HCY metabolism and related factors are displayed in Table 2. We found that the folic acid supplementation and adequate folic acid supplementation during periconception were associated with a reduced risk of early spontaneous abortion (OR = 0.50, 95% CI = 0.38–0.65; OR = 0.44, 95% CI = 0.35–0.54). The serum folate deficiency and higher plasma HCY in early pregnancy were associated with a higher risk of early spontaneous abortion (OR = 5.87, 95% CI = 1.53–22.50; OR = 2.94, 95% CI = 1.14–7.57). The women who carried the MTHFR 677TT genotype were associated with a higher risk of early spontaneous abortion in comparison to the women carrying the CC/CT genotype (OR = 2.32, 95% CI = 1.20–4.50). There was no association between early spontaneous abortion and the serum vitamin B_12_ deficiency.

**Table 2:** The association between early spontaneous abortion and HCY metabolism and related factors.

	Case	Control	Model 1^a^		Model 2^b^	
	*n* (%)	*n* (%)	OR (95% CI)	*p*	OR (95% CI)	*p*
Folic acid supplementation during periconception						
Yes	379 (75.80)	862 (86.20)	0.42 (0.31–0.58)	<0.001	0.50 (0.38–0.65)	<0.001
No	121 (24.20)	138 (13.80)	1.00		1.00	
Adequate folic acid supplementation during periconception						
Yes	200 (40.00)	603 (60.30)	0.38 (0.29–0.49)	<0.001	0.44 (0.35–0.54)	<0.001
No	300 (60.00)	397 (39.70)	1.00		1.00	
Serum folate deficiency						
No	182 (91.00)	392 (98.00)	1.00		1.00	
Yes	18 (9.00)	8 (2.00)	4.85 (1.45–16.15)	0.012	5.87 (1.53–22.50)	0.010
Serum vitamin B_12_ deficiency						
No	164 (82.00)	350 (87.50)	1.00		1.00	
Yes	36 (18.00)	50 (12.50)	1.54 (0.79–2.97)	0.200	1.10 (0.49–2.46)	0.826
Higher plasma HCY						
No	170 (85.00)	378 (94.50)	1.00		1.00	
Yes	30 (15.00)	22 (5.50)	3.03 (1.34–6.88)	0.006	2.94 (1.14–7.57)	0.025
MTHFR677 genotype						
CC /CT	138 (69.00)	334 (83.50)	1.00		1.00	
TT	62 (31.00)	66 (16.50)	2.27 (1.29–4.00)	0.004	2.32 (1.20–4.50)	0.013

^a^Unadjusted.

^b^Adjusted for maternal age, maternal education, residence and household wealth index.

### The effects of interaction of HCY metabolism and related factors on early spontaneous abortion

The effects of interaction of HCY metabolism and related factors on early spontaneous abortion are displayed in Table 3. In terms of the risks for early spontaneous abortion, there was no multiplicative interaction between higher plasma HCY in early pregnancy, serum folate deficiency in early pregnancy, maternal MTHFR 677TT genotype and no folic acid supplementation during periconception (OR = 2.35, 95% CI: 0.39–14.09). There was an interaction between higher plasma HCY in early pregnancy, serum folate deficiency in early pregnancy and maternal MTHFR 677TT genotype (OR = 1.76, 95% CI: 1.17–4.03). There was an interaction between higher plasma HCY and serum folate deficiency in early pregnancy (OR = 3.46, 95% CI: 2.49–4.81), and there was an interaction between serum folate deficiency in early pregnancy and maternal MTHFR 677TT genotype (OR = 3.50, 95% CI: 2.78–5.18). The above interactions are all synergistic.

**Table 3:** The effects of interaction of HCY metabolism and related factors on early spontaneous abortion.

	OR (95% CI)	*p*
Higher plasma HCY * Serum folate deficiency * MTHFR 677TT genotype * No folic acid supplementation during periconception	2.35 (0.39–14.09)	0.351
Higher plasma HCY * Serum folate deficiency * MTHFR 677TT genotype	**1.76 (1.17–4.03)**	**0.017**
Higher plasma HCY * No folic acid supplementation during periconception * MTHFR 677TT genotype	0.42 (0.01–12.24)	0.614
Higher plasma HCY * No folic acid supplementation during periconception * Serum folate deficiency	2.03 (0.40–10.40)	0.395
Serum folate deficiency * No folic acid supplementation during periconception * MTHFR 677TT genotype	4.08 (0.93–16.23)	0.066
Higher plasma HCY * Serum folate deficiency	**3.46 (2.49–4.81)**	**<0.001**
Higher plasma HCY * MTHFR 677TT genotype	0.85 (0.42–1.71)	0.644
Serum folate deficiency * MTHFR 677TT genotype	**3.50 (2.78–5.18)**	**<0.001**

Adjusted for maternal age, maternal education, residence and household wealth index.

The bold values indicate that it is easy to find statistically significant results.

### The pathway analysis of HCY metabolism and related factors affecting early spontaneous abortion

The logit test method of generalized structural equation model (GSEM) was used to construct the pathway diagram of HCY metabolism and related factors affecting early spontaneous abortion. By modifying the model, the test results of the fit which was the confirmed pathway diagram of the final model are displayed in the Table 4. Root Mean Square Error of Approximation (RMSEA), Normed Fit Index (NFI), Comparative Fit Index (CFI), Parsimony-adjusted Normed Fit Index (PNFI) and Parsimony-adjusted Comparative Fit Index (PCFI) were all close to the fit standards. It indicated that the model had a good adaptability and was an acceptable model.

**Table 4:** The test results of the final model fit.

Test statistic	Fit standard	Test result	Judgment of model fit
RMSEA	<0.089 (<0.05 excellent)	0.061	good
NFI	>0.9	0.902	excellent
CFI	>0.9	0.905	excellent
PNFI	>0.5	0.471	good
PCFI	>0.5	0.472	good

**Figure 2:**
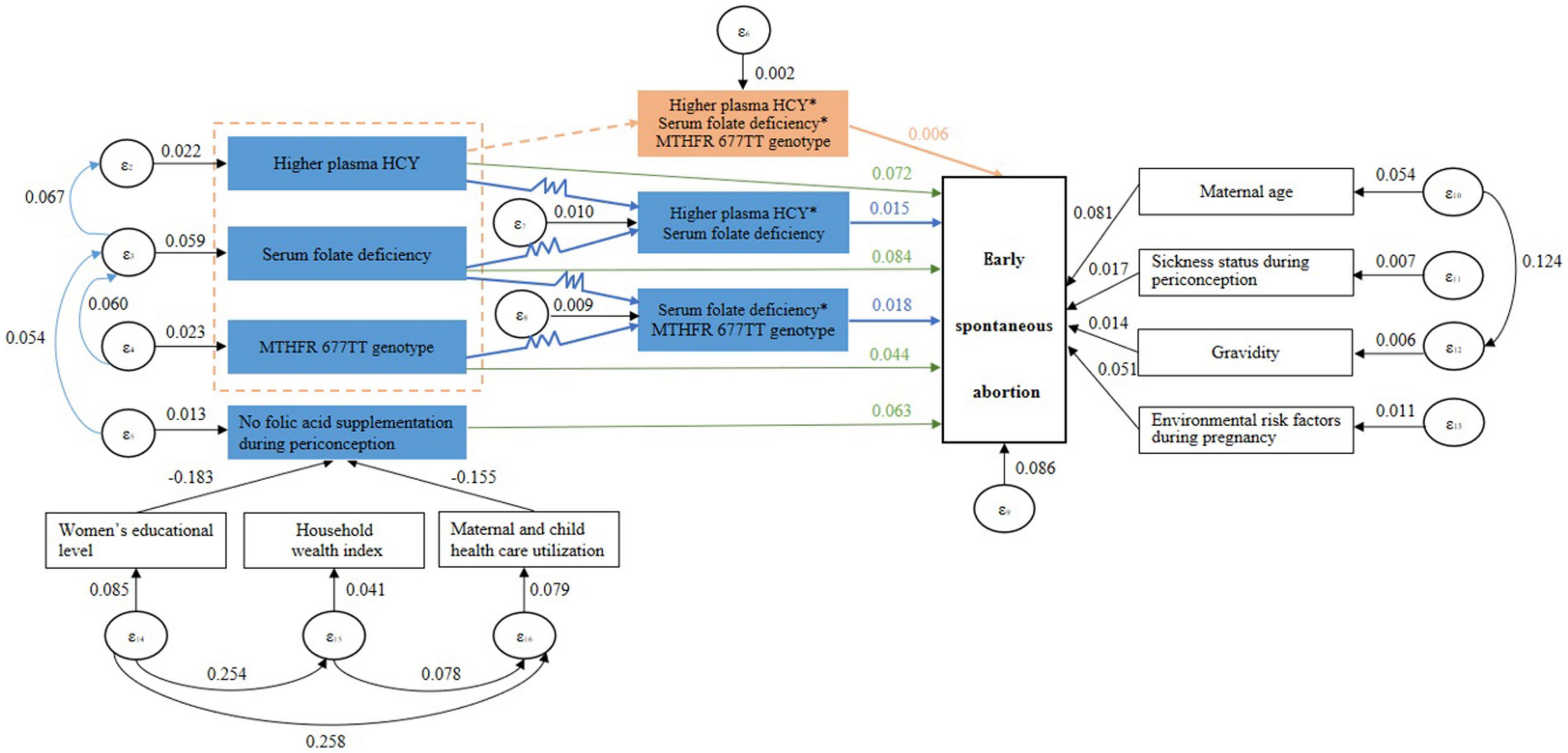
The pathway diagram of HCY metabolism and related factors affecting early spontaneous abortion.

The results of pathway analysis showed that women’s educational level and maternal and child health care utilization affected the occurrence of early spontaneous abortion by influencing the folic acid supplementation during periconception. The folic acid supplementation during periconception affected the occurrence of early spontaneous abortion by influencing the level of serum folate or plasma HCY in early pregnancy. The maternal MTHFR 677 gene polymorphism affected the occurrence of early spontaneous abortion by influencing the level of serum folate in early pregnancy. In terms of the risks for early spontaneous abortion, there was an interaction between higher plasma HCY in early pregnancy, serum folate deficiency in early pregnancy and maternal MTHFR 677TT genotype, and there was an interaction between higher plasma HCY and serum folate deficiency in early pregnancy, and there was an interaction between serum folate deficiency in early pregnancy and maternal MTHFR 677TT genotype. The direction of the above interactions was all positive, which showed the synergistic effect. The occurrence risk of early spontaneous abortion was significantly increased if multiple factors existed at the same time. The pathway diagram are displayed in Figure 2.

## Discussion

There are very few studies on the pathway analysis of HCY metabolism and related factors affecting early spontaneous abortion. Most studies only analyzed the association between a certain factor and spontaneous abortion, or studied the association between the related factors of HCY metabolism and other adverse pregnancy outcomes. A meta-analysis reported MTHFR C677T and A1298C were significantly associated with some types of congenital defects and adverse pregnancy outcomes (21). Another study from America found that the combined effect of elevations in maternal homocysteine, smoking, and the MTHFR 677\u00B0C > T polymorphism increase the risk of having a CHD-affected pregnancy (22).

Our study presents that folic acid supplementation and adequate folic acid supplementation during periconception were the protective factors of early spontaneous abortion. The serum folate deficiency, higher plasma HCY in early pregnancy, the women who carried the MTHFR 677TT genotype were the risk factors of early spontaneous abortion. The serum vitamin B_12_ deficiency is not associated with the risk of early pregnancy loss. The majority of previous studies conducted in this regard did not have stringent inclusionexclusion criteria and the results presented were variable. A study from India found that folic acid deficiency is not associated with the risk of early pregnancy loss, and vitamin B_12_ deficiency and elevated homocysteine levels are independent risk factors for early pregnancy loss, and of higher risk when combined (23). Other related studies showed the association of polymorphisms in important genes of folate metabolism such as methylenetetrahydrofolate reductase (MTHFR 677C > T, 1298A > C) with recurrent pregnancy loss (24, 25). This suggests that genetic susceptibility and nutritional deficiency of vitamin B_12_ may be synergistically contributing to the risk of early pregnancy loss. Our study has showed the similar conclusion that there was an synergistic interaction between higher plasma HCY in early pregnancy, serum folate deficiency in early pregnancy and maternal MTHFR 677TT genotype in terms of the risks for early spontaneous abortion. Therefore, more research is needed in the future to investigate the effects of folic acid and vitamin B_12_ on early pregnancy loss.

B vitamins play the important roles in HCY metabolic pathway, providing key metabolites for HCY metabolism and DNA methylation. Folic acid, also known as vitamin B_9_, is involved in the metabolism of tetrahydrofolate (THF) and HCY as a methyl donor. It is also important for DNA synthesis, repair and methylation (26). Vitamin B_2_, B_6_ and B_12_ which are important coenzymes or coenzyme factors in HCY metabolic pathway except folate affect HCY metabolism and DNA methylation by regulating HCY metabolic pathway. Vitamin B_6_ is a cofactor of serine hydroxymethyltransferase in the conversion of THF to 5,10-methylene tetrahydrofolate (5,10-methylene THF). Vitamin B_2_ is a coenzyme factor which MTHFR reduces 5,10-methylene THF to 5-methyltetrahydrofolate (5-methyl THF). Vitamin B_12_ which is precursor of methionine synthase (MTR) is involved in the metabolism of HCY. The MTHFR gene which is the folate metabolism gene is also involved in HCY metabolism and affects methylation level. It’s the most common mutation that the 677th base cytosine of the MTHFR gene mutate into thymine. The mutated MTHFR gene which would lead to a disease in MTHFR activity can block the HCY metabolic pathway. HCY mainly affects the occurrence of spontaneous abortion in two ways. On the one hand, the higher level of HCY would put cells at the state of high oxidative stress and have embryotoxicity, which may result in dysplastic embryo and spontaneous abortion (27). On the other hand, the higher level of HCY would damage vascular endothelial cells and disrupt the balance of the coagulation-anticoagulation system by stimulating the production and release of free radicals, and that might lead to placental embolism and spontaneous abortion due to accelerating the formation of placental thrombosis (28). In addition, DNA methylation in HCY metabolism is important for embryo development. During mammalian embryo implantation and development, DNA methylation undergoes dramatic reprogramming that is crucial for the development of both the embryo and the maternal endometrium (29–32). A study found that inhibition of DNA methylation maintenance led to a decreased implantation rate of embryos, increased fetal absorption, and poor fetal and placental development (33). Therefore, further experiments are needed to clarify changes in gene expression and methylation states of genes under the DNA methylation maintenance defect in early pregnancy loss pathogenesis. There have been other studies investigating the association between a certain factor of HCY metabolism and spontaneous abortion. A study in Syria found the low serum vitamin B_12_ increased the risk of recurrent abortion (34). A meta-analysis reported that high HCY levels in both plasma and serum as well as low folate levels in serum and red blood cells are significantly associated with risk of recurrent spontaneous abortion (35). A study in Sweden found that low plasma folate levels were associated with an increased risk of early spontaneous abortion (36). A study from America reported that use of vitamin supplements during early pregnancy was associated with reduced odds of miscarriag (37). A study in Chongqing, China reported that maternal periconceptional folic acid supplementation is associated with a reduced risk of spontaneous abortion (38). In fact, all results from these studies suggested that a certain factor of HCY metabolism was associated with the occurrence of spontaneous abortion.

However, some limitations of our study merit discussion. First, due to the observational study, we cannot rule out the possible limitation of recall bias. Second, this study can only preliminarily verify the association between early spontaneous abortion and HCY metabolism, and cannot confirm the causal relationship. Third, because of the lack of other maternal biomarkers about HCY metabolism, such as vitamin B_2_, vitamin B_6_ and lncRNA-H19, the relationship between HCY metabolism and the risk of spontaneous abortion cannot be further analyzed. Finally, residual confounding cannot be ruled out despite the careful consideration of potential confounders.

## Conclusion

In conclusion, our findings suggest that folic acid supplementation and adequate folic acid supplementation during periconception were the protective factors of early spontaneous abortion. The serum folate deficiency, higher plasma HCY in early pregnancy, the women who carried the MTHFR 677TT genotype were the risk factors of early spontaneous abortion. The women’s educational level and maternal and child health care utilization affected the occurrence of early spontaneous abortion by influencing the folic acid supplementation during periconception. The folic acid supplementation during periconception affected the occurrence of early spontaneous abortion by influencing the level of serum folate or plasma HCY in early pregnancy. The maternal MTHFR 677 gene polymorphism affected the occurrence of early spontaneous abortion by influencing the level of serum folate in early pregnancy. In terms of the risks for early spontaneous abortion, there was an interaction between higher plasma HCY in early pregnancy, serum folate deficiency in early pregnancy and maternal MTHFR 677TT genotype, and there was an interaction between higher plasma HCY and serum folate deficiency in early pregnancy, and there was an interaction between serum folate deficiency in early pregnancy and maternal MTHFR 677TT genotype. The direction of the above interactions was all positive, which showed the synergistic effect. The occurrence risk of early spontaneous abortion was significantly increased if multiple factors existed at the same time.

## Data availability statement

The original contributions presented in the study are included in the article/Supplementary material. The data collection was done by the research team and the data was kept by the project group. Requests to access the datasets should be directed to the corresponding author.

## Ethics statement

The study was conducted in accordance with the Declaration of Helsinki, and the Xi’an Jiaotong University Health Science Center approved it (approval code: 2012008). The participants provided their written informed consents to participate in this study.

## Author contributions

FL: Writing – original draft, Software, Methodology, Investigation, Formal analysis, Conceptualization. LZ: Writing – original draft, Software, Data curation. LW: Writing – review & editing, Visualization, Investigation. WW: Writing – review & editing, Resources, Validation. FW: Writing – review & editing, Supervision, Resources, Funding acquisition, Conceptualization.
