# Supplementary material for: Association between early spontaneous abortion and homocysteine metabolism
Source: Front Med (Lausanne). 2024 Mar 25;11:1310112. doi: 10.3389/fmed.2024.1310112 (PMC10999573; doi:10.3389/fmed.2024.1310112)
Supplement: Supplementary file 1 [file Table_1.docx]

**Supplementary Table S1**

Comparison Table of English Abbreviations

| **English Abbreviations** | **Full Name in English** |
| --- | --- |
| DHFR | Dihydrofolate Reductase |
| DHF | Dihydrofolate |
| THF | Tetrahydrofolate |
| MTHFR | Methylene Tetrahydrofolate Reductase |
| MTR | Methionine Synthase |
| MTRR | Methionine Synthase Reductase |
| SAHH | S-Adenosyl-L-Homocysteine Hydrolase |
| SAH | S-Adenosylhomocysteine |
| SAM-DM | S-adenosylmethionine-dependent methyltransferase |
