# Supplementary material for: Association between early spontaneous abortion and homocysteine metabolism
Source: Front Med (Lausanne). 2024 Mar 25;11:1310112. doi: 10.3389/fmed.2024.1310112 (PMC10999573; doi:10.3389/fmed.2024.1310112)
Supplement: Supplementary file 2 [file Table_2.docx]

**Supplementary Table S2**

General characteristics of mothers included in the subgroup for the assessment of HCY metabolism-related biomarkers and those not included in the subgroup

| **Characteristics** | **Mothers included in the subgroup [n(%)]**  **(*N*=600)** | **Mothers not included in the subgroup [n(%)]**  **(*N*=900)** | ***P*** |
| --- | --- | --- | --- |
| Maternal age |  |  | 0.510 |
| <30 years | 378(63.00) | 582(64.67) |  |
| ≥30 years | 222(37.00) | 318(35.33) |  |
| Maternal education |  |  | 0.817 |
| Junior high school or below | 57(9.50) | 93(10.33) |  |
| Senior high school/Secondary specialized school | 124(20.67) | 191(21.22) |  |
| College degree or above | 419(69.83) | 616 (68.45) |  |
| Residence |  |  | 0.654 |
| Rural | 259(43.17) | 378(42.00) |  |
| Urban | 341(56.83) | 522(58.00) |  |
| Household wealth index |  |  | 0.929 |
| Poor | 215(35.83) | 328(36.44) |  |
| Moderate | 184(30.67) | 279(31.00) |  |
| Rich | 201(33.50) | 293(32.56) |  |
| Environmental risk factors during pregnancy |  |  | 0.540 |
| Yes | 317(52.83) | 490(54.44) |  |
| No | 283(47.17) | 410(45.56) |  |
| Gravidity |  |  | 0.641 |
| 1 | 211(35.17) | 306(34.00) |  |
| ≥2 | 389(64.83) | 594(66.00) |  |
| Parity |  |  | 0.332 |
| 0 | 318(53.00) | 454(50.44) |  |
| ≥1 | 282(47.00) | 446(49.56) |  |
| Sickness status during periconception |  |  | 0.111 |
| Yes | 393(65.50) | 553(61.44) |  |
| No | 207(34.50) | 347(38.56) |  |
| Maternal and child health care utilization |  |  | 0.517 |
| Worse | 492(82.00) | 726(80.67) |  |
| Better | 108(18.00) | 174(19.33) |  |
