# Supplementary material for: Association between early spontaneous abortion and homocysteine metabolism
Source: Front Med (Lausanne). 2024 Mar 25;11:1310112. doi: 10.3389/fmed.2024.1310112 (PMC10999573; doi:10.3389/fmed.2024.1310112)
Supplement: Supplementary file 4 [file Presentation_1.pdf]

Supplementary Method section "sample size calculations"

The estimated percentages about pregnant women in the sample size calculations were based on the data between the birth defects group and the control group in our previous survey in Shaanxi China[1,2]. For the association between homocysteine (HCY) metabolism and related factors and early spontaneous abortion, on the assumptions of the estimated percentages of adequate folic acid supplementation during periconception in cases and controls being 37.1% and 45.4% respectively, the correlation of exposure between cases and controls being zero, the type I error rate 0.05, and the power of the test 80%, the sample sizes of cases and controls were 412 and 824 respectively. A study in China found that the minor allele frequency of the MTHFR C677T in the Chinese Han population was 40%[3]. For the association between HCY metabolism related blood biochemical markers and early spontaneous abortion, on the assumptions of the estimated incidence of exposure factors of these women in cases and controls being 53% and 40% respectively, the correlation of exposure between cases and controls being zero, the type I error rate 0.05, and the power of the test 80%, the sample sizes of cases and controls were 172 and 344 respectively. Considering 20% unresponsive research subjects, the final analysis included 500 cases and 1000 controls who had completed the questionnaires, which met the sample size requirement.

References

[1] Pei L, Kang Y, Cheng Y, et al. The Association of Maternal Lifestyle with Birth Defects in Shaanxi Province, Northwest China[J]. PloS One, 2015, 10 (9): e0139452.

[2] Yang J, Cheng Y, Pei L, et al. Maternal iron intake during pregnancy and birth outcomes: a cross-sectional study in Northwest China[J]. British Journal of Nutrition, 2017, 117 (6): 862-871.

[3] Yang B, Liu Y, Li Y, et al. Geographical distribution of MTHFR C677T, A1298C and MTRR A66G gene polymorphisms in China: findings from 15357 adults of Han nationality[J]. PloS One, 2013, 8 (3): e57917.
